# Dosimetric comparison between proton beam therapy and photon radiation therapy for locally advanced esophageal squamous cell carcinoma

**DOI:** 10.1186/s13014-018-0966-5

**Published:** 2018-02-09

**Authors:** Yasuhiro Hirano, Masakatsu Onozawa, Hidehiro Hojo, Atsushi Motegi, Sadatomo Zenda, Kenji Hotta, Shunsuke Moriya, Hidenobu Tachibana, Naoki Nakamura, Takashi Kojima, Tetsuo Akimoto

**Affiliations:** 1 Division of Radiation Oncology and Particle Therapy, National Cancer Center Hospital East, 6-5-1, Kashiwanoha, Kashiwa, Chiba, 277-8577 Japan; 2 Division of Gastroenterology and Gastrointestinal Oncology, National Cancer Center Hospital East, 6-5-1, Kashiwanoha, Kashiwa, Chiba, 277-8577 Japan

**Keywords:** Proton beam therapy, IMRT, 3DCRT, Locally advanced esophageal cancer, In silico dose distribution

## Abstract

**Background:**

The purpose of this study was to perform a dosimetric comparison between proton beam therapy (PBT) and photon radiation therapy in patients with locally advanced esophageal squamous cell carcinoma (ESCC) who were treated with PBT in our institution. In addition, we evaluated the correlation between toxicities and dosimetric parameters, especially the doses to normal lung or heart tissue, to clarify the clinical advantage of PBT over photon radiation therapy.

**Methods:**

A total of 37 consecutive patients with Stage III thoracic ESCC who had received PBT with or without concurrent chemotherapy between October 2012 and December 2015 were evaluated in this study. The dose distributions of PBT were compared with those of dummy 3-dimensional conformal radiation therapy (3DCRT) and Intensity Modulated Radiation Therapy (IMRT), focusing especially on the doses to organs at risk, such as normal lung and heart tissue.

**Results:**

Of the 37 patients, the data from 27 patients were analyzed. Among these 27 patients, four patients (15%) developed grade 2 pericardial effusion as a late toxicity. None of the patients developed grade 3 or worse acute or late pulmonary and cardiac toxicities. When the dosimetric parameters between PBT and planned 3DCRT were compared, all the PBT domestic variables for the lung dose except for lung V10 GyE and V15 GyE were significantly lower than those for the dummy 3DCRT plans, and the PBT domestic variables for the heart dose were also significantly lower than those for the dummy 3DCRT plans. When the PBT and IMRT plans were compared, all the PBT domestic variables for the doses to the lung and heart were significantly lower than those for the dummy IMRT plans. Regarding the correlation between the grades of toxicities and the dosimetric parameters, no significant correlation was seen between the occurrence of grade 2 pericardial effusion and the dose to the heart.

**Conclusions:**

When the dosimetric parameters of the dose distributions for the treatment of patients with locally advanced stage III ESCC were compared between PBT and 3DCRT or IMRT, PBT enabled a significant reduction in the dose to the lung and heart, compared with 3DCRT or IMRT.

## Background

One standard treatment option for stage II-III esophageal squamous cell carcinoma (ESCC) is neoadjuvant chemotherapy followed by surgical resection, and the 5-year overall survival rate is reported to be 36.8%–61% [1–3]. Chemoradiotherapy (CRT) is also a curative-intent non-surgical treatment option for resectable ESCC, especially for patients who refuse surgical resection or are unsuited for surgical resection. The results of the Japan Clinical Oncology Group (JCOG) 9906, a Phase II study that evaluated CRT for the patients with stage II-III ESCC, showed an initial complete response rate of 62.2% and a 5-year survival rate of 36.8% [4]. However, 4 (5.3%) related deaths occurred because of late toxicities including pneumonitis (*n* = 2), pericarditis (*n* = 1), and pleural effusion (*n* = 1). Long-term cardiopulmonary toxicities sometimes caused life-threatening events or death in patients who have received CRT, and the main causes for the development of cardiopulmonary toxicities are excessive radiation doses to normal lung and heart tissue, in addition to the combination of chemotherapy with radiation therapy. Therefore, it is important to reduce the incidence and severity of late toxicities, since this would lead to an improved quality of life (QOL) for patients who are able to achieve disease control.

Particle therapy including proton beam therapy (PBT) has a unique physical characteristic, called the Bragg peak, and can deliver a high dose to the tumor while sparing the surrounding normal tissues. An in silico dose distribution comparison between photon radiation therapy and PBT for esophageal cancer shows that proton beam therapy has clear therapeutic advantages, especially a dose reduction to at-risk organs, over conventional external radiotherapy [5]. Based on these backgrounds, we have been applying PBT with concurrent chemotherapy for the treatment of patients with ESCC since 2012. The purpose of the present study was to perform a dosimetric comparison between PBT and photon radiation therapy in patients who were treated with PBT for locally advanced esophageal ESCC in our institution. We also evaluated the correlation between the grade and/or incidence of toxicities and dosimetric parameters, especially the doses to normal lung or heart tissue, to clarify the clinical advantages of PBT over photon radiation therapy.

## Methods

### Patients

Approval for this study was obtained from the National Cancer Center Institutional Review Board. Consecutive patients with thoracic ESCC who received PBT with or without concurrent chemotherapy between October 2012 and December 2015 were enrolled as candidates for this study. Among these, we selected 37 consecutive patients with Stage III thoracic ESCC for dosimetric comparison between PBT and photon radiation therapy because the rationale for the coverage of the radiation field, including the elective nodal region, differs according to disease stage, and this might have resulted in differences in the doses to risk organs, such as the lung and heart.

### Pretreatment evaluation

Clinical staging was based on the American Joint Committee on Cancer staging, 7th edition. The staging evaluations included a barium swallow test, an endoscopy examination of the esophagus and stomach, and a computed tomography examination of the neck, chest, and abdomen. When necessary, 18F-fluorodeoxyglucose positron emission tomography was also performed.

### Treatment details

The chemotherapy regimen consisted of 2 cycles of cisplatin (CDDP) (70 mg/m^2^) on day 1 and the continuous infusion of fluorouracil (FU) (700 mg/m^2^) on days 1 to 4 repeated every 4 weeks, 2 cycles of CDDP (75 mg/m^2^) on day 1 and the continuous infusion of FU (1000 mg/m^2^) on days 1 to 4 repeated every 4 weeks, 2 cycles of nedaplatin (80-90 mg/m^2^) on day 1 and the continuous infusion of FU (800 mg/m^2^) on days 1 to 5 repeated every 4 weeks, or FU (700 mg/m^2^) on days 1 to 4 repeated every 4 weeks, with concurrent PBT. The fractionated schema for PBT was 60 GyE in 30 fractions over 6 weeks. Dose reduction, the postponement of either drug or the discontinuation of PBT was implemented to prevent a worsening of adverse events.

For the PBT simulation, all the patients were placed in a supine position. Simulation CT images were obtained in 3-mm thick slices. An in-house treatment planning system with a calculation grid size of 1.876 mm was used for the PBT treatment planning. The dose calculations were performed using the pencil beam dose calculation algorithm. The gross tumor volume (GTV) was defined as the primary tumor and clinically positive lymph nodes. The clinical target volume (CTV) encompassed the GTV and the subclinical tumor extension. For primary lesion, 2 cm expansion cranio-caudally and 3 mm expansion radially, and no CTV margin was added for nodal disease. The planning target volume (PTV) covered the CTV with a 5-mm in all directions. A total dose of 60 GyE in 30 fractions was given to the primary tumor and the metastatic lymph nodes. The dose constraints for the organs at risk (OARs) were as follows: normal lung V20 GyE (percentage of the normal lung volume irradiated with more than 20 GyE) < 20%, heart V30 GyE (percentage of the heart volume irradiated with more than 30 GyE) < 46% and mean dose < 26 GyE, and a spinal cord Dmax (maximum point dose) of 48 GyE. The beam output was modulated with a relative biologic effectiveness (RBE) of 1.1, based on a previous animal examination [6].

### Dosimetric analysis

The dosimetric data for the OARs were collected from each treatment plan. For normal lung tissue, the V20, 15, 10, and 5 GyE and the mean dose were calculated. For the heart, the V40, 30, 20, and 10 GyE and the mean dose were calculated. For the spinal cord, the maximum point dose was calculated. Conformity index (CI) determined as the volume of the 90% prescription isodose surface divided by PTV was also evaluated. To compare the dosimetric parameters of the dose distribution between PBT and 3-dimensional conformal radiation therapy (3DCRT) and between PBT and intensity-modulated radiation therapy (IMRT), dummy 3DCRT plans and IMRT plans were created using the same simulated computed tomography (CT) images, structures and dose prescription settings for PBT. 3DCRT, IMRT and PBT plans were normalized to cover 50% of the PTV with 100% of the prescription dose.

In the dummy IMRT planning, the plan was approved when 50% of the PTV received 100% of the prescribed dose (D50 = 100% dose prescription), after eliminating hot spots receiving ≥110% of the prescribed dose. Dose constraints for the normal lung (V20 Gy), the heart (mean dose), and the spinal cord (maximum dose) were set at < 30%, < 50 Gy, and < 45 Gy, respectively. All the plans were generated using 5-treatment beams with 6-MV X-rays. IMRT delivery was performed using the sliding window technique with the Varian Millennium 120-MLC. AAA was used as the dose calculation engine.

The dummy 3DCRT and IMRT plans were created using the Xio version 5.0 (Elekta, Sweden) and the Eclipse version 11.0 treatment planning system (Varian Medical System, USA), respectively. In the dummy 3DCRT plans, photon energies of 6 -MV and 10 -MV were used. For the dose calculation of the 3DCRT plan, the superposition method with inhomogeneous correction was used, with a grid size of 2 mm. The plans were approved when the CTV could receive at least 95% without exceeding the dose constraints for the OARs, as described previously.

### Toxicity assessment

Adverse events caused by PBT with or without concurrent chemotherapy were evaluated based on the Common Terminology Criteria for Adverse Events (CTCAE), version 4.0. In this study, non-hematological toxicities including pulmonary and cardiac adverse events were mainly evaluated. Acute adverse events were assessed within 2 months from the first day of PBT and were graded based on the worst symptoms experienced during this period. Symptoms or events caused by PBT and that occurred at least 3 months after the first day of PBT were evaluated as late adverse events.

### Statistical analysis

The survival analysis was performed by the Kaplan-Meier method. Overall survival was calculated from the first day of PBT to death due to any cause. Progression-free survival was defined as the time from the first day of PBT to that of disease progression, evidence of residual disease or death from any cause.

The statistical analysis was performed using IBM SPSS Statistics 22. A paired *t*-test was performed to compare the doses to the OARs.

## Results

### Patient characteristics

Of the 37 patients, data from 27 patients were analyzed retrospectively. Ten of the 37 patients were excluded from the study because 3 patients received a total dose of 66 GyE in 33 fractions, two patients received a total dose of 50.4 GyE in 28 fractions, and five patients received a combination of x-rays and PBT. The characteristics of the 27 patients who were included in the analysis are summarized in Table 1. Of these 27 patients, three patients received PBT alone because two patients refused to receive chemotherapy and one patient was not a candidate for chemotherapy because of a poor physical condition. These patients didn’t receive any chemotherapy including neoadjuvant or adjuvant setting.

**Table 1:** Patient characteristics

Characteristic	Patients (*n* = 27)	(%)
Male	23	85.2
Female	4	14.8
Age (y)		
Range	50-90	
Median	70	
T stage		
T3	25	92.6
T4	2	7.4
N stage		
N0	0	0
N1	16	59.3
N2	10	37.0
N3	1	3.7
Stage		
IIIA	15	55.6
IIIB	9	33.3
IIIC	3	11.1
Location of the lesion		
Ut	5	18.5
Mt	9	33.3
Mt./Lt	6	22.2
Lt	5	18.5
Lt/Ae	2	7.4
Chemotherapy		
5-Fu + CDDP	16	59.3
5-Fu + nedaplatin	7	25.9
5-Fu	1	3.7
Radiotherapy alone	3	11.1

*Abbreviations*: *Ut* upper thoracic esophagus, *Mt.* middle thoracic esophagus, *Lt* lower thoracic esophagus, *Ae* abdominal esophagus, *Fu* fluorouracil, *CDDP* cisplatin

### Survival

The median follow-up period was 442 days (range, 69-1, 468 days) for all the eligible patients. The median follow-up period for the censored cases was 445 days (range, 69-1, 468 days). The overall survival and progression-free survival at 1 year after completion of PBT were 90.8 and 40.6%, respectively.

### Toxicities

Acute and late pulmonary and cardiac adverse events are shown in Table 2. One case required an interruption in PBT because of grade 3 esophagitis however, none of the patients required PBT interruption because of pulmonary or cardiac adverse events. Four patients (15%) developed grade 2 pericardial effusion as a late toxicity. The median period from the last day of PBT until the development of grade 2 pericardial effusion was 179 days, ranging from 56 to 280 days. At the time of analysis, none of the patients had developed grade 3 or worse, acute or late, pulmonary or cardiac toxicities.

**Table 2:** Acute and late pulmonary and cardiac adverse events

	Grade1	Grade2	Grade3	Grade4-5
Acute toxicity				
Pneumonitis	7 (26%)	0	0	0
Pericardial effusion	–	1 (4%)	0	0
Late toxicity				
Pneumonitis	17 (63%)	0	0	0
Pleural effusion	5 (19%)	0	0	0
Pericardial effusion	–	4 (15%)	0	0

### Dosimetric analysis

**Fig. 1:**
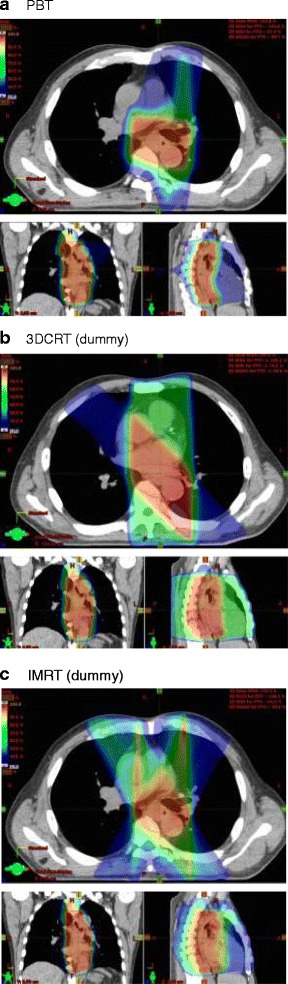
Axial, sagittal, and coronal views of the dose distributions for T3 N1 middle thoracic esophageal cancer. **a** PBT, **b** dummy plan for 3DCRT using 4 portals, **c** dummy plan for IMRT. The red line represents the PTV. PBT, proton beam therapy; 3DCRT, 3-dimensional conformal radiation therapy; IMRT, intensity-modulated radiation therapy; PTV, planning target volume

Dummy plans for 3DCRT at the same radical dose as PBT were generated for nine patients using foue portal beams, while those of the other 18 patients were generated using more than four portal beams because of the difficulty in creating an acceptable dose to the spinal cord arising from the undesirable spatial relationship between the extent of the disease and the spinal cord. A representative PBT dose distribution, the dummy plan for 3DCRT, and the dummy plan for IMRT for a patient with T3 N1 middle thoracic esophageal cancer is shown in Fig. 1.

A paired *t*-test was performed to compare the dosimetric results between the PBT plans and the 3DCRT plans and between the PBT plans and the IMRT plans statistically (Table 3). When the dosimetric parameters between the PBT and 3DCRT plans were compared, all the PBT domestic variables regarding the lung dose except for the lung V10 GyE and V15 GyE were significantly lower than those of the dummy 3DCRT plans (Table 3). In addition, all the PBT domestic variables regarding the heart dose (mean dose, V20, V30, V40) were significantly lower than those of the dummy 3DCRT plans. When the dosimetric parameters were compared between the PBT and IMRT plans, all the PBT domestic variables regarding the dose to the lung and heart including the lung V10 and V15 GyE were significantly lower than those of the dummy IMRT plans (Table 3).

**Table 3:** Dosimetric comparison between PBT and 3DCRT, PBT and IMRT

End point	DVH parameters	Technique	Mean	SD	SE mean	*p* value. (vs. 3DCRT)	*p* value. (vs. IMRT)
Lung	V20 (%)	PBT	11.6607	5.18604	.99805	.001	< 0.01
		3DCRT	16.9652	5.64638	1.08665		
		IMRT	17.7941	5.19620	1.00001		
	V15 (%)	PBT	17.8696	7.22321	1.39011	.052	.006
		3DCRT	21.6615	6.75645	1.30028		
		IMRT	22.9630	5.64911	1.08717		
	V10 (%)	PBT	21.3307	9.07254	1.74601	.058	< 0.01
		3DCRT	25.7222	7.46624	1.43688		
		IMRT	30.0181	6.84990	1.31826		
	V5 (%)	PBT	25.2478	10.77897	2.07441	.005	< 0.01
		3DCRT	33.0893	8.79872	1.69331		
		IMRT	45.1811	10.52645	2.02582		
	Mean dose (Gy)	PBT	5.8322	2.24148	.43137	< 0.01	< 0.01
		3DCRT	8.2111	2.14654	.41310		
		IMRT	9.4778	2.17052	.41772		
Heart	V40 (%)	PBT	16.3059	11.42262	2.19828	< 0.01	0.012
		3DCRT	48.1837	21.03337	4.04787		
		IMRT	26.5770	16.91562	3.25541		
	V30 (%)	PBT	22.0252	14.45506	2.78188	< 0.01	< 0.01
		3DCRT	56.1574	21.49217	4.13617		
		IMRT	50.7126	23.40755	4.50479		
	V20 (%)	PBT	38.4963	23.91227	4.60192	< 0.01	< 0.01
		3DCRT	64.1178	23.03079	4.43228		
		IMRT	69.3259	26.97548	5.19143		
	Mean dose (Gy)	PBT	17.6089	9.68207	1.86331	< 0.01	.001
		3DCRT	30.9844	11.45297	2.20412		
		IMRT	9.4778	2.17052	.41772		
Spinal cord	Maximum dose (Gy)	PBT	38.1400	4.96680	.95586	< 0.01	.155
		3DCRT	47.2896	2.22998	.42916		
		IMRT	39.7119	2.64873	.50975		
CI		PBT	1.8796	.21319	.04103	< 0.01	< 0.01
		3DCRT	3.1886	.61704	.11875		
		IMRT	1.6243	.23493	.04521		

*SD* standard deviation, *SE* standard error, *CI* conformity index

### Correlation between toxicities and dosimetric parameters

Regarding the correlation between the grades and/or incidence of toxicities and the dosimetric parameters, we evaluated the impact of the dose to the heart on the occurrence of grade 2 pericardial effusion, since four patients developed grade 2 pericardial effusion as a late toxicity and none of the patients developed grade 3 or severer late toxicities. However, no significant correlation between the occurrence of grade 2 pericardial effusion and the dose to heart, including the mean heart dose, heart V20, V30 and V40, was observed, although all the parameters in the patients who developed grade 2 pericardial effusion were slightly higher than those in the patients who did not (Table 4).

**Table 4:** Correlation between late pericardial effusion toxicity and dose to heart

DVH parameters	Pericardial Effusion (Grade)	Mean	SD	SE mean	*p* value
V40 (%)	2	16.2850	13.53387	6.76694	.997
	0	16.3096	11.36759	2.37031	
V30 (%)	2	23.2550	19.36524	9.68262	.858
	0	21.8113	13.98136	2.91531	
V20 (%)	2	42.2300	32.64928	16.32464	.742
	0	37.8470	22.96571	4.78868	
Mean dose (Gy)	2	19.1400	13.02880	6.51440	.739
	0	17.3426	9.33480	1.94644	

## Discussion

When the dosimetric parameters between PBT and the 3DCRT or IMRT plans were compared, the present results demonstrated that PBT resulted in a significantly lower dose to the lung and heart, while providing an optimal dose to the treatment targets, compared with 3DCRT or IMRT for locally advanced stage III ESCC. In radiotherapeutic management for ESCC, concurrent CRT has been established as a standard treatment modality for patients with ESCC who are not suited to undergo surgical resection or who refuse surgery, and satisfactory clinical outcomes have been obtained [7–9]. However, late toxicities such as pleural effusion or pericardial effusion have been important issues that should be solved.

Table 5 summarizes the reported results of late toxicities after CRT in esophageal cancer [4, 10–14]. The main cardiopulmonary late toxicities were pleural effusion, pericardial effusion and radiation pneumonitis, and the incidences of grade 3 or worse pericardial effusion and radiation pneumonitis were around 10% and 3%, respectively. However, the occurrence of cardiopulmonary late toxicities impairs the QOL of patients, although the symptoms are usually improved by optimal medical interventions. In addition, several authors have described that cardiopulmonary complications sometimes become life-threatening [11, 13]. Ishikura et al. reported that two patients died because of acute myocardial infarction among 78 patients who achieved complete remission, and the cause of death of 8 patients might have been related to cardiopulmonary toxicity [11]. Morota et al. also reported that one patient died of heart failure among 69 patients treated with CRT for esophageal cancer [13]. Considering these clinical outcomes, the occurrence of severe late toxicities should be reduced and avoided. (Here is the location of Table 5.)

**Table 5:** Late cardiopulmonary toxicities Grade 2 or greater after CRT for esophageal carcinoma on previous reports

Auther Reference year	N	Souce	Radiation dose modality	Radiation techniques	Pleural effusion (%)		Pericardial effusion (%)		Radiation pneumonitis (%)		Other toxicities
					Gr. 2	Gr. 3-4	Gr. 2	Gr. 3-4	Gr. 2	Gr. 3-4	–
Kato(4) (2011)	76	RTOG/EORTC late radiation morbidity scoring scheme	60 Gy 3DCRT	A-P opposed (initial), bilateral oblique (boost)	6.6	9.2	6.6	15.8	7.9	4.2	–
Kato(10) (2013)	51	CTCAE v. 3.0	50.4 Gy 3DCRT	Three or four fields	14	0	12	0	51	6	–
Ishikura(11) (2003)	78	RTOG/EORTC late radiation morbidity scoring scheme	60 Gy 3DCRT	A-P opposed (initial), bilateral oblique or multiple (boost)	9	10	10	10	1.3	3.8	Heart Failure Grade4: 2.6% Myocardial Infarction Grade5: 2.6%
Kumekawa(12) (2006)	34	NCI-CTC v. 2.0 RTOG/EORTC late radiation morbidity scoring scheme	60 Gy 3DCRT	A-P opposed (initial), bilateral oblique (boost)	5.9	8.9	8.9	8.9	0	2.9	Heart Failure Grade4: 8.9% Cardiac ischema Grade3: 2.9% Grade4: 5.9%
Morota(13) (2009)	69	RTOG/EORTC late radiation morbidity scoring scheme	60 Gy 3DCRT	A-P opposed (initial), bilateral oblique or multiple (boost)	13	1.5	–	–	7.2	1.5	Heart Failure Grade2: 1.5% Grade5: 1.5% Cardiac ischema Grade3: 1.5% Pericarditis Grade3: 1.5%
Kumar(14) (2012)	23	CTCAE v. 3.0	50 or 50.4 Gy 3DCRT	Anterior and two lateral beams (initial), anterior and two oblique beams (boost)	–	–	–	–	74	17	–

*Abbreviations*: *Gr.* Grade, *A-P* anterior-posterior, *CTCAE* common terminology criteria for adverse events, *RTOG* radiation therapy oncology group, *EORTC* European Organization for Research and Treatment of Cancer, *NCI-CTC* National Cancer Institute Common Toxicity Criteria, *N* number of the patients assessed for late toxicities

As described above, the main reasons for the development of pulmonary and/or cardiac late toxicities is thought to be an excessive dose to OARs such as the heart and lung, and the intensification of treatment through combined concurrent chemotherapy also affects the occurrence of severe pulmonary and/or cardiac late toxicities. However, the intensity of CRT must be maintained to obtain satisfactory clinical outcomes, including the initial response and long-term outcomes, because combined concurrent chemotherapy has been established as a standard treatment for locally advanced ESCC. Thus, reducing the radiation dose to the lung and heart would be an important and effective approach to decreasing the severity or incidence of treatment-related toxicities caused by CRT.

The current standard beam arrangement of 3DCRT for esophageal cancer is 4 beams, especially with a heavier weighting in the AP/PA direction, and this increases the dose to the heart and spinal cord. To reduce the dose to OARs, a multi-portal technique has been tested, and an improvement in the incidence of severe late cardiopulmonary toxicities has been obtained. Kato et al. reported in a study of CRT for stage II-III ESCC using a multi-field technique that late cardiopulmonary toxicities included grade 3 pneumonitis (5.9%), but none of the cases had cardiac adverse events of grade 3 or worse severity [10]. Thus, the application of the multi-field technique was useful for reducing the dose to the heart.

An alternative, effective approach to reducing the dose to OARs is the application of particle therapy, especially PBT, combined with chemotherapy. Charged particles such as PBT and heavy particles have Bragg peak properties, meaning that almost all of their energy can be deposited in the target volume with tissues beyond the tumor location receiving minimal doses [15]. Considering the anatomical relationship between the esophagus and the heart/lungs, the application of particle therapy is likely to reduce the dose to OARs, compared with photon radiation therapy. Actually, in a study comparing PBT with photon radiation therapy for esophageal cancer evaluated according to the tumor control probability (TCP) and the normal tissue complication probability (NTCP), the PBT plans were able to reduce the doses to structures of the lung and heart better like this study and appeared to have clear therapeutic advantages over photon radiation therapy [5]. Zhang et al. compared 4-dimensional computed tomography-based treatment plans with PBT or IMRT for distal esophageal cancer, and the application of PBT resulted in a more significant dose reduction to the lung than IMRT; however, no improvement in the dose to the heart was seen [16]. A limitation of the study by Zhang et al. was the small number of patients who were analyzed (15 patients); furthermore, the analysis was limited to patients with distal esophageal cancer, since the main histological type in the United States is adenocarcinoma, which usually develops in the distal esophagus. This difference in the locations of the primary tumors would affect the dose to OARs, such as the heart and lungs, because of the anatomical relationship between the esophagus and surrounding OARs. Therefore, the results of this study are expected to provide valuable information regarding the efficacy of PBT for patients with thoracic esophageal squamous cell carcinoma.

Regarding the incidence and severity of cardiopulmonary toxicities, the results of the current study showed a much lower incidence than those reported by other authors (Table 5). As described above, the main reason for this difference is likely the reduced dose to OARs that can be achieved using PBT. Actually, no significant correlation between the occurrence of grade 2 pericardial effusion and the dose to the heart, including the mean heart dose, was seen in this study, mainly because of the limited number of patients who developed grade 2 or severer toxicities. The further accumulation of data is warranted to clarify the correlation between the grade of toxicity and the dosimetric parameters of PBT.

The results of this study demonstrated the efficacy of PBT with concurrent chemotherapy for locally advanced ESCC based on a comparison of the dosimetric parameters to OARs between PBT and 3DCRT or IMRT plans. However, the present study had several limitations. First, the follow-up duration was not sufficient to evaluate long-term late toxicities. Second, the number of patients enrolled in this study was too small to evaluate the impact of the location of primary tumors on the dose to OARs. However, a strength of this study is that the analyzed patients were limited to consecutive patients with Stage III thoracic esophageal cancer, whose treatment strategies in terms of radiation field coverage were relatively uniform. Therefore, we believe that this study suggests that PBT with concurrent chemotherapy might be an effective treatment option for locally advanced esophageal squamous cell cancer. Based on the results of the dosimetric analysis, we are now conducting a phase I dose-escalating study of PBT with concurrent full-dose chemotherapy for patients with stage IB-III esophageal squamous cell carcinoma. In the future, large-scale prospectively randomized studies should be conducted to clarify the effectiveness of PBT with concurrent chemotherapy for the treatment of locally advanced esophageal cancer.

## Conclusions

The results of this study demonstrated that PBT enabled a significant reduction in the dose to the lung and heart, compared with treatment plans for 3DCRT or IMRT, for locally advanced stage III ESCC when the dosimetric parameters between PBT and 3DCRT or IMRT plans were compared.

## Abbreviations

3DCRT: 3-dimensional conformal radiation therapy
CDDP: Cisplatin
CRT: Chemoradiotherapy
CTV: Clinical target volume
ESCC: Esophageal squamous cell carcinoma
FU: Fluorouracil
GTV: Gross tumor volume
GyE: Gray equivalent
IMRT: Intensity-modulated radiation therapy
OARs: Organs at risk
PBT: Proton beam therapy
PTV: Planning target volume
